# Gender disparities in childhood vaccination in India: exploring the role of son preference using NFHS-5 data

**DOI:** 10.3389/fpubh.2025.1709865

**Published:** 2026-01-23

**Authors:** Soumen Barik, Dewaram A. Nagdeve, Alex Motes Carvalho, Mayank Singh

**Affiliations:** 1 Department of Fertility & Social Demography, International Institute for Population Sciences (IIPS), Mumbai, India; 2 Department of Epidemiology and Biostatistics, KAHER, Belagavi, Karnataka, India

**Keywords:** ANC visits, childhood immunization, Fairlie decomposition, GLM, higher birth order, son preference

## Abstract

**Background:**

Every child has the fundamental human right to life-saving healthcare, including vaccination. Yet in India, this right is less accessible to girls in households shaped by son preference, a deep-rooted cultural bias that devalues girl children. While immunization prevents millions of deaths each year, girls are less likely to be fully vaccinated—not due to biological need, but because of gender-based discrimination in the allocation of time and care within the home.

**Materials and methods:**

Using nationally representative data from the National Family Health Survey-5 (NFHS-5; 2019–21), we analyzed a sample of 20,899 girls aged12–23 months. Full vaccination (BCG, three doses of DPT, three doses of polio, measles) was the outcome. Maternal son preference was the key exposure. We employed a four-stage generalized linear model (GLM) with a log link to estimate adjusted relative risks, progressively controlling for child, maternal, and household variables. Fairlie decomposition analysis was conducted to quantify the extent to which these characteristics explain the observed vaccination gap between girls and boys.

**Results:**

Girls in son-preferring households had significantly lower vaccination rates (70.8% vs. 76.9%). The GLM showed a consistent negative association between son preference and vaccination across all models; however, it became non-significant (ARR: 0.99, 95% CI: 0.97–1.02) after full adjustment. Fairlie decomposition analysis revealed that 84% of this gap was statistically explained by factors such as birth order (21%), antenatal care (ANC) visits (29%), and household wealth (21%), which were the largest contributors. The remaining 16% was unexplained by unmeasured cultural norms.

**Conclusion:**

Interventions should target girls of higher birth order, those in the poorest households, and those born to mothers with low education. Strategies could include leveraging antenatal care visits to deliver gender-sensitive health messaging and expanding the role of community health workers (CHWs) and Anganwadi teams for door-to-door monitoring. Closing the remaining 16% of the unexplained gap demands confronting cultural norms, ensuring that every child—regardless of birth order or family wealth—has an equal right to protection.

## Introduction

Son preference—the desire for a boy child over a girl child—is a deeply entrenched cultural and social phenomenon that has been documented globally as a driver of gender inequality and discrimination against girls (1, 2). This preference for male children often manifests in the unequal allocation of household resources, including access to healthcare, prenatal care, nutrition, and education (3, 4). Vaccination coverage is a critical aspect of child health, as it trains the immune system to recognize and fight specific pathogens (5). Son preference has significant negative consequences and is a key determinant of girls’ health and access to disease prevention. Vaccination protects children from life-threatening illnesses such as measles, polio, and diphtheria; however, disparities in immunization rates between boys and girls have been observed in regions with strong son preference (6, 7). Son preference is not just an attitude but a structural driver of gender inequity, rooted in broader systems of social stratification. The social determinants of health framework describes it as a cultural and power-based barrier shaping health access (8). Gender norms theory explains how internalized beliefs devalue girls’ health, skewing parental care decisions. Together, these theories show how son preference intersects with poverty, birth order, and maternal factors to reduce girls’ vaccination coverage (3).

Immunization saves an estimated 3.5–5 million lives each year by protecting against diseases such as diphtheria, tetanus, pertussis, influenza, and measles (9). It is among the most essential and cost-effective strategies for reducing childhood illness and mortality (10). Vaccination disparities are particularly pronounced in South Asia, where son preference is deeply rooted in cultural and economic practices (11, 12). Many child deaths in India are preventable through immunization. However, vaccination coverage remains low, especially among girls, due to inequitable access (13). Research has shown that son preference can lead to discriminatory practices in healthcare access, with girls often receiving delayed or incomplete vaccinations compared to boys (14, 15). Girls born in India have a 40% higher likelihood of experiencing poor health compared to boys and are less likely to receive healthcare, particularly immunization (16, 17). This disparity is particularly pronounced in rural areas and among economically disadvantaged families, where resource constraints and agricultural dependence exacerbate existing biases (18, 19).

For example, studies have found that girls of higher birth order are less likely to receive timely vaccinations compared to boys, reflecting patterns of son preference observed in India (8). Furthermore, cultural norms that prioritize boys’ health over girls’ can result in lower prioritization of girls’ vaccination needs, leaving them vulnerable to preventable diseases (20, 21). The consequences of inadequate vaccination coverage for girls are severe, contributing to higher morbidity and mortality rates among girl children (22, 23). For instance, studies from central, northern, eastern, and northeastern Indian states have shown that girls are disproportionately affected by vaccine-preventable diseases such as measles and pertussis, which can have long-term health implications (24, 25). Similarly, global studies have highlighted how gender disparities in vaccination contribute to broader inequities in child survival and development (26, 27).

Addressing these disparities is not only a matter of public health but also a critical step toward achieving gender equality and fulfilling global commitments, such as the Sustainable Development Goals (SDGs) (53). Vaccination is a fundamental right for every child (28); however, son preference continues to undermine this right for millions of girls (29). Much of the research on son preference has focused on gender differences in child mortality, imbalanced sex ratios at birth, and disparities in access to health and nutrition, while relatively less attention has been given to its role in driving gender disparities in childhood immunization (8). While significant research has examined spatial heterogeneity in vaccination coverage, the predictors of vaccination inequality, and vaccine hesitancy (30–33), the role of maternal son preference in contributing to gender gaps in immunization remains largely underexplored.

By revealing these disparities, this research can inform policies that promote gender-equitable vaccination coverage, ensuring that every child, regardless of gender, has access to life-saving interventions. This analysis aimed to explore the relationship between son preference and girls’ vaccination status using National Family Health Survey-5 (NFHS-5) data. By examining patterns of vaccine uptake among girls, we sought to understand how son preference drives disparities in immunization coverage. In addition, this study investigated regional and socioeconomic factors that may either mitigate or exacerbate these inequities. Understanding these dynamics is crucial for designing targeted interventions that promote equitable healthcare access and ensure that all children, regardless of gender, receive the protection they need against preventable diseases. This study also aimed to challenge harmful gender norms, advocate for universal immunization programs, and work toward a more inclusive society where every child has an equal opportunity to thrive.

## Materials and methods

### Data source

The present study utilized unit-level data from the fifth round of the National Family Health Survey (NFHS-5) conducted in India in 2019–21. The NFHS-5 is a nationally representative, cross-sectional household survey that employs a stratified two-stage sampling design, covering 36 states/union territories and 707 districts across the country. The survey collects detailed information on demographic, health, and nutrition indicators for women aged 15–49 years and their children under 5 years of age. The sampling frame was based on the 2011 Census of India. NFHS datasets are publicly available through the Demographic and Health Surveys (DHS) website after a simple user registration, without requiring special permission. In our study, son preference was measured by comparing a woman’s actual and desired sex composition. Women who reported a higher desired number of sons than daughters were classified as having son preference.

**Figure 1:**
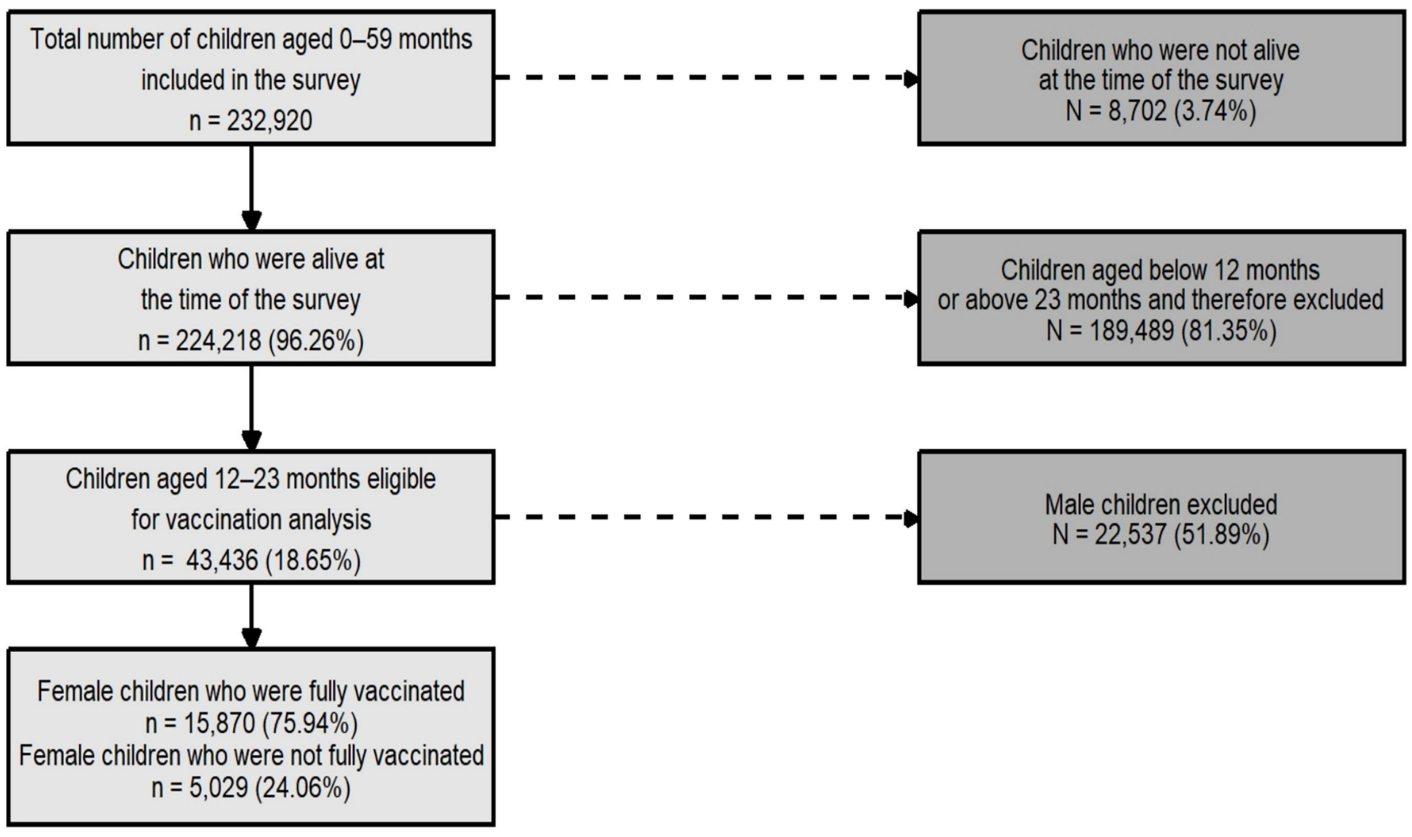
Flow diagram of sample selection showing inclusion criteria for girls aged 12–23 months.

As depicted in Figure 1, the study sample was drawn from 2,32,920 children aged 0–59 months enumerated in the NFHS-5. After excluding 3.74% (*n* = 8,702) of deceased children, 2,24,218 children were alive at the time of the survey. Of these, 43,436 were aged 12–23 months. For the present analysis, 20,899 girl children aged 12–23 months were selected to examine son preference and gender disparities in childhood vaccination coverage.

### Outcome variables

The primary dependent variable was vaccination status among girl children aged 12–23 months, defined according to the fifth round of the National Family Health Survey (2019–2021). This variable was binary, with ‘1’ indicating ‘Fully Vaccinated’ and ‘0’ indicating ‘Not fully vaccinated’.
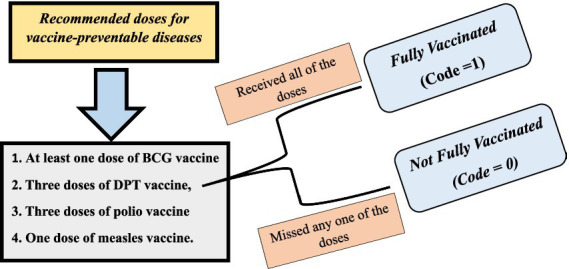


### Explanatory variables

This study considered independent variables related to vaccination status among girl children aged 12–23 months. The selected independent variables were categorized into child characteristics, maternal characteristics, and household/community-level factors, as shown in the Table 1 below.

**Table 1:** Description of independent variables used in the study.

Variables	Categories
Child characteristics	
Age of the child in months	12–15, 16–19, and 20–23
Birth order	First, second or third, and fourth and above
Size of the child at birth	Small, average, and large
Birth weight	Low birth weight and optimal birth weight
Maternal factors	
Maternal son preference	No son preference and son preference
Mother’s age in years	15–24, 25–34, and 35 and above
Mother’s education	No education, primary, secondary, and higher
ANC visit	Three or fewer and more than three
Delivery place	Non-institutional and Institutional
Maternal BMI	Underweight, normal, and overweight or obese
Pregnancy	Wanted and unwanted
Household and community-level variables	
Exposure to mass media	No and any
Distance to health facility	No problem and problem
Exposed to family planning messages	Not exposed and exposed
Household members	1–4, 5–8, and more than 8
Sex of the household head	Male and female
Wealth index	Poorest, poorer, middle, richer, and richest
Social caste	SC/ST, OBC, and other
Religion	Hindu, Muslim, Christian, and other
Residence	Urban and rural

SC/ST, Scheduled Caste/Scheduled Tribe; OBC, Other Backward Class.

### Statistical analysis

Descriptive statistics were used to estimate the prevalence of full vaccination among girl children, stratified by maternal son preference and other covariates. Bivariate associations were examined using the chi-squared test to identify significant predictors. To assess the relationship between son preference and vaccination status, a generalized linear model (GLM) with a log link and binomial family was employed, producing relative risks (RRs) with 95% confidence intervals. A four-stage GLM with a binomial family and log link was used to obtain RRs instead of odds ratios, as the high prevalence of full vaccination could cause logistic regression models to exaggerate effect sizes. A log-link GLM therefore offered a more interpretable, risk-based measure suitable for public health translation. Model adequacy was assessed using the variance inflation factor (VIF < 5; Obtained VIF < 3.7) to rule out multicollinearity, and a link test was conducted to evaluate appropriate model specification, demonstrating no evidence of misspecification. We also compared nested specifications progressively (Model 1 through Model 4) to examine the stability of effect estimates across child, maternal, and household/community covariates, and the results remained directionally consistent. To further quantify the contribution of explanatory variables to differences in full vaccination coverage by son preference, a Fairlie decomposition analysis was conducted, partitioning the gap into explained and unexplained components. All analyses accounted for the complex design of the NFHS-5 by applying sampling weights and were conducted using Stata v17 (StataCorp LLC, College Station, TX).

### Mathematical equation of the Fairlie decomposition

The Fairlie decomposition for a binary outcome, such as vaccination of girls in households with and without son preference, is given by the following equation:


$${\bar{Y}}_{W}-{\bar{Y}}_{B}=\sum_{k}[\frac{1}{N_{W}}\sum_{i=1}^{N_{W}}F(X_{\mathrm{Wi}}\hat{\beta })-\frac{1}{N_{B}}\sum_{i=1}^{N_{B}}F(X_{\mathrm{Bi}}\hat{\beta })]$$

Where,

${\bar{Y}}_{W}\;$ = $\begin{array}{l} \text{Average predicted probability of full}\;\\ \text{vaccination without}\;\mathrm{son}\;\text{preference} \end{array}$

${\bar{Y}}_{B}=$ $\begin{array}{l} \text{Average predicted probability of full vaccination}\;\\ \text{in households with}\;\mathrm{son}\;\text{preference} \end{array}$

$N_{W}\;\text{and}\;N_{B}=$ Sample sizes of thetwogroups

$X_{\mathrm{Wi}}\;\text{and}\;X_{\mathrm{Bi}}=$ $\begin{array}{l} \text{Vectors of explanatory variables}\;\\ \text{for each}\;i\;\text{in groups}\;W\;\text{and}\;B \end{array}$

$\hat{\beta }=$ Estimated coefficients from the logistic regression model

F(.)= Cumulative distribution function of the logistic model

Following Fairlie (2005) (54), the total difference was decomposed into explained and unexplained components as follows:


$${\bar{Y}}_{W}-{\bar{Y}}_{B}=\{\text{Explained component}\}+\{\text{Unexplained component}\}$$

$$\begin{array}{l} {\bar{Y}}_{W}-{\bar{Y}}_{B}=\{\frac{1}{N_{W}}\sum_{i=1}^{N_{W}}F(X_{\mathrm{Wi}}{\hat{\beta }}_{W})-\frac{1}{N_{B}}\sum_{i=1}^{N_{B}}F(X_{\mathrm{Bi}}{\hat{\beta }}_{W})\}+\{\frac{1}{N_{B}}\sum_{i=1}^{N_{B}}F(X_{\mathrm{Bi}}{\hat{\beta }}_{W})-\frac{1}{N_{B}}\sum_{i=1}^{N_{B}}F(X_{\mathrm{Bi}}{\hat{\beta }}_{B})\} \end{array}$$

Here, the explained component represents the portion of the vaccination gap attributable to differences in observed characteristics, including child, maternal, and household factors. The unexplained component reflects differences due to the effects of these characteristics or unobserved factors.

Each model was developed in four stages, as shown in the Table 2 below:

**Table 2:** Description of sequential adjustment models used in the analysis.

Model	Child factors	Maternal factors	Household factors
Model 1	Unadjusted Model		
Model 2	Adjusted	Not adjusted	
Model 3	Adjusted		Not adjusted
Model 4	Fully adjusted		

Note: Color is used just to differentiate the adjusted and unadjusted.

### Ethical consideration

This study was based on the fifth round of the National Family Health Survey (NFHS) conducted from 2019 to 2021. The NFHS-5 is a publicly available secondary dataset collected by the International Institute for Population Sciences (IIPS), Mumbai, under the supervision of the Ministry of Health and Family Welfare, Government of India. All primary data collection, including participant recruitment and informed consent, was conducted by the IIPS following ethical approval from the IIPS Institutional Review Board (IRB) and the ICF Institutional Review Board. The authors analyzed only de-identified, publicly available data obtained from the DHS program website; therefore, no additional ethical approval was required for this secondary analysis.

## Results

Table 3 depicts the distribution of vaccination status among girl children aged 12–23 months according to background characteristics. All characteristics were significantly associated with vaccination status, as indicated by the chi-squared test (*p* < 0.05) with a 95% confidence interval. Complete vaccination among girl children was higher (76.66%) among older children aged 20–23 months, whereas almost 78.67% of first-birth order children were fully vaccinated. Girl children with an average size at birth and optimal birth weight were also more likely to be fully vaccinated, with coverage rates of 76.82 and 78.01%, respectively. A majority (76.47%) of girl children born to mothers aged 25–34 years were fully vaccinated compared to those born to younger and older mothers. Nearly 67.11% of children born to mothers with no education were the least likely to be fully vaccinated compared to children of mothers with formal education. Higher completion of girl child vaccination was observed among mothers with more than three antenatal care (ANC) visits (70.43%), individuals who delivered in a health institution (77.30%), mothers who were overweight or obese (79.52%), mothers in the age group of 20–29 years at first birth (77.39%) and mothers who reported the pregnancy as wanted (76.53%). Inequalities in girl child vaccination were also observed at the household and community levels. Vaccination completion was higher among households exposed to mass media (78.30%), those reporting no difficulty in accessing healthcare facilities due to distance (77.90%), and those receiving family planning messages (78.15%). Households with fewer members (1–4) reported a higher rate of complete girl child vaccination (77.73%) compared to households with more than four members. Households in the poorest wealth quintile had lower complete vaccination coverage (69.8%). By religion, Christian families reported higher complete vaccination among girl children (79.39%). Urban and rural households showed an identical prevalence of complete vaccination (75%).

**Table 3:** Distribution of vaccination status among girl children aged 12–23 months by background characteristics (*N* = 20,899).

Background characteristics	Not fully vaccinated (*n* = 5,029)	Fully vaccinated (*n* = 15,870)	Chi squared *p*-value
Age of the child			
12–15 months	1841 (24.66)	5,461 (75.34)	<0.001
16–19 months	1,696 (24.17)	5,253 (75.83)	
20–23 months	1,492 (23.34)	5,156 (76.66)	
Birth order			
First order	1738 (21.33)	6,475 (78.67)	<0.001
Second/third	2,435 (24.25)	7,688 (75.75)	
Forth and above	856 (33.23)	1707 (66.77)	
Size of the child at birth			
Small	567 (24.13)	1,695 (75.87)	0.001
Average	3,370 (23.18)	11,269 (76.82)	
Large	966 (26.22)	2,803 (73.78)	
Birth weight			
Low birth weight	1775 (23.36)	5,869 (76.64)	<0.001
Optimal birth weight	2,486 (21.99)	9,094 (78.01)	
Maternal characteristics			
Women’s age (years)			
15–24 years	1971 (24.10)	6,244 (75.90)	0.001
25–34 years	2,643 (23.53)	8,556 (76.47)	
>=35 years	415 (28.65)	1,070 (71.35)	
Women’s education			
No education	1,302 (32.89)	2,820 (67.11)	<0.001
Primary	647 (24.74)	1835 (75.26)	
Secondary	2,463 (21.76)	8,691 (78.24)	
Higher	617 (20.87)	2,524 (79.13)	
ANC visits			
<=3 ANC visits	2,481 (29.57)	5,661 (70.43)	<0.001
>3 ANC visits	2,101 (19.75)	8,975 (80.25)	
Place of delivery			
Non-institutional	985 (37.12)	1,494 (62.88)	<0.001
Institutional	4,044 (22.70)	14,376 (77.30)	
Maternal BMI			
Normal	2,722 (23.44)	8,529 (76.56)	0.003
Underweight	994 (25.70)	3,055 (74.30)	
Overweight/obese	608 (20.48)	2,238 (79.52)	
Age of the mother at first birth			
<=20 years	1798 (26.94)	4,738 (73.06)	<0.001
20–29 years	3,079 (22.61)	10,554 (77.39)	
30–49 years	152 (24.21)	578 (75.79)	
Pregnancy wanted			
Wanted	4,492 (23.47)	14,540 (76.53)	<0.001
Unwanted	537 (29.72)	1,330 (70.28)	
Household and community characteristics			
Exposure to mass media			
No	1861 (30.13)	4,111 (69.87)	<0.001
Any	3,168 (21.70)	11,759 (78.30)	
Distance to health facility			
No problem	1,696 (22.10)	6,109 (77.90)	<0.001
Problem	3,333 (25.42)	9,761 (74.58)	
Exposure to family planning messages			
Not exposed	1798 (30.30)	3,918 (69.70)	<0.001
Exposed	3,231 (21.85)	11,952 (78.15)	
Household members			
1–4	1,303 (22.27)	4,267 (77.73)	<0.001
5–8	2,800 (23.45)	9,159 (76.55)	
More than 8	926 (28.90)	2,444 (71.10)	
Household head			
Male	4,248 (23.96)	13,485 (76.04)	<0.001
Female	781 (24.70)	2,385 (75.30)	
Wealth index			
Poorest	1,685 (30.17)	3,828 (69.83)	<0.001
Poorer	1,258 (25.52)	3,646 (74.48)	
Middle	817 (20.21)	3,230 (79.79)	
Richer	710 (21.25)	2,872 (78.75)	
Richest	559 (20.95)	2,294 (79.05)	
Social caste			
SC/ST	2,150 (24.47)	6,295 (75.53)	<0.001
OBC	1817 (23.19)	6,228 (76.81)	
Other	780 (24.52)	2,655 (75.48)	
Religion			
Hindu	3,418 (23.28)	11,993 (76.72)	<0.001
Muslim	829 (28.35)	2,108 (71.65)	
Christian	563 (20.61)	1,129 (79.39)	
Other	219 (23.71)	640 (76.29)	
Residence			
Urban	1,003 (24.26)	3,302 (75.74)	<0.001
Rural	4,026 (24.00)	12,568 (76.00)	

Totals may not sum to *N* = 20,899 due to missing values and the use of weighted NFHS-5 estimates.

**Figure 2:**
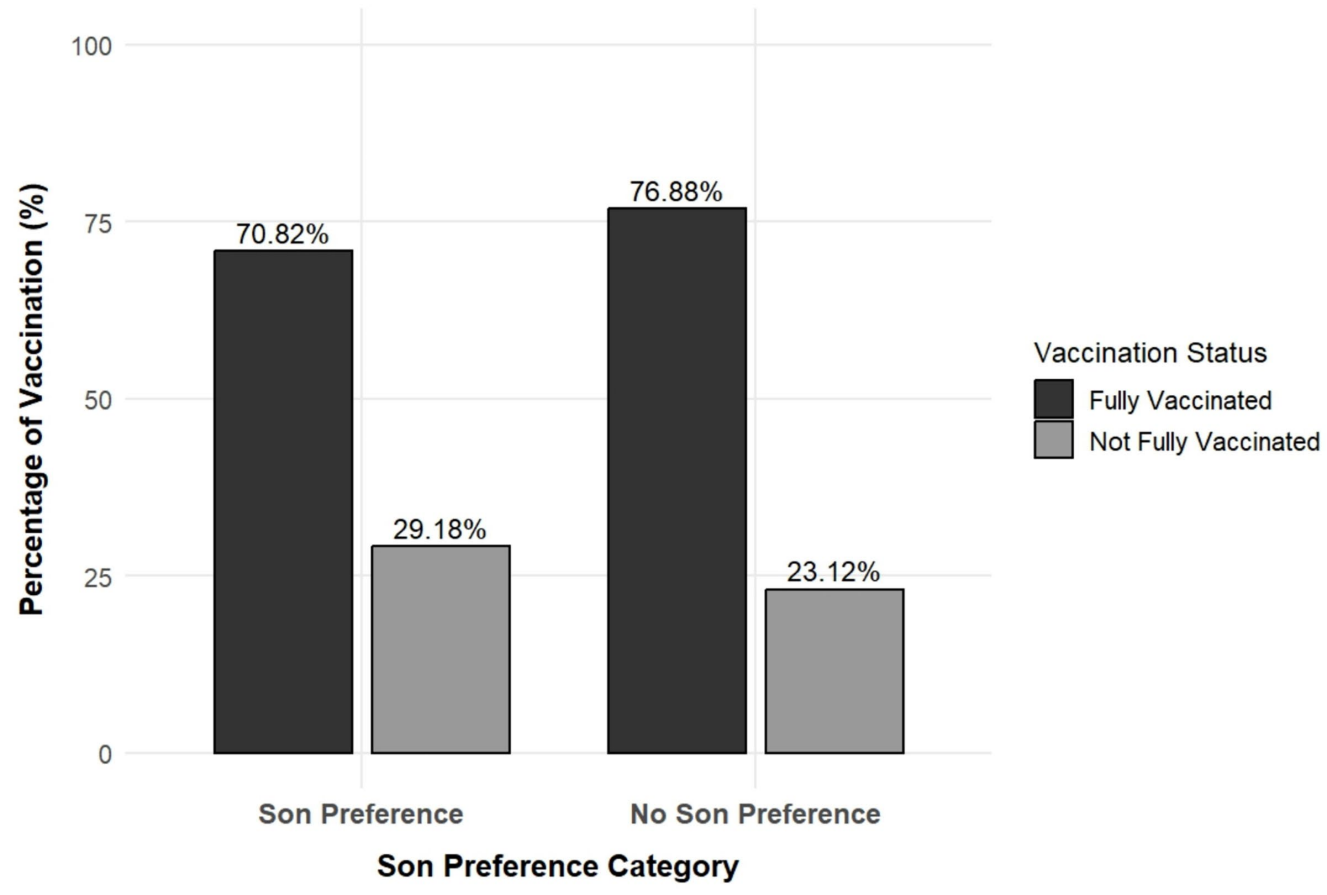
Prevalence of full vaccination among girl children by maternal son preference (*N* = 20,899). The prevalence of vaccination was calculated using weighted percentage distributions only.

Figure 2 depicts the influence of son preference on girl child vaccination. Households without son preference had a higher prevalence of full vaccination among girls (76.88%) compared to households with son preference (70.82%).

Table 4 shows that maternal son preference was associated with lower completion of girl child vaccination. According to unadjusted Model 1, the likelihood of complete girl child vaccination was lower among households with son preference (URR: 0.93; 95% CI: 0.91–0.95) compared to households with no son preference. Similar lower odds of complete girl child vaccination were observed among households with son preference, even after controlling for child, maternal, household, and community-level variables. In adjusted Model 2, which accounted for child-level factors, younger age in months, higher birth order, average size at birth, and low birth weight were associated with reduced vaccination coverage. In adjusted Model 3, which accounted for child and maternal factors, girl children born to mothers with unwanted pregnancies had a lower likelihood of complete vaccination (ARR: 0.79; 95% CI: 0.77–0.82), whereas older, highly educated mothers with adequate ANC visits and institutional deliveries were positively associated with complete girl child vaccination. In adjusted Model 4, which accounted for child, maternal, household, and community factors, exposure to mass media and family planning messages, smaller household size, higher household wealth, and rural residence were associated with higher odds of complete girl child vaccination.

**Table 4:** Generalized linear model estimates of maternal son preference and girl child vaccination in India (NFHS-5).

Background characteristics	Model 1	Model 2	Model 3	Model 4
Son preference of women	URR (95% CI)	ARR	ARR	ARR
No son preference				
Son preference	0.93*** (0.91, 0.95)	0.95*** (0.93, 0.98)	0.98*** (0.99, 0.96)	0.99 (0.97, 1.02)
Age of the child				
12–15 months				
16–19 months		1.00 (0.99, 1.02)	1.00*** (1.00, 1.00)	1.00 (0.98, 1.02)
20–23 months		1.03** (1.01, 1.05)	1.01*** (1.01, 1.01)	1.02* (1.00, 1.04)
Birth order				
First				
Second/third		0.98** (0.96, 0.99)	0.99*** (0.99, 0.99)	0.98 (0.96, 1.00)
Forth and above		0.90*** (0.87, 0.92)	0.75*** (0.72, 0.77)	0.94** (0.90, 0.97)
Size of the child at birth				
Small				
Average		1.01 (0.99, 1.04)	1.00*** (1.00, 1.00)	1.00 (0.97, 1.03)
Large		0.98 (0.95, 1.01)	0.99*** (0.99, 0.99)	0.99 (0.95, 1.02)
Birth weight				
Low birth weight				
Optimal birth weight		1.02** (1.01, 1.04)	1.01*** (1.01, 1.01)	1.015 (1.00, 1.03)
Maternal characteristics				
Women’s age (years)				
15–24 years				
25–34 years			1.01*** (1.01, 1.01)	1.01 (0.99, 1.04)
>=35 years			1.01*** (1.01, 1.01)	1.03 (0.98, 1.07)
Women’s education				
No education				
Primary			1.02 (1.02, 1.02)	1.04* (1.01, 1.08)
Secondary			1.02*** (1.02, 1.02)	1.05** (1.02, 1.08)
Higher			1.02*** (1.02, 1.02)	1.01 (0.97, 1.05)
ANC visits				
<=3 ANC visits				
>3 ANC visits			1.03 (1.03, 1.03)	1.09*** (1.07, 1.11)
Place of delivery				
Non-institutional				
Institutional			1.03 (1.03, 1.03)	1.06* (1.01, 1.10)
Maternal BMI				
Normal				
Underweight			1.00*** (1.00, 1.00)	1.00 (0.98, 1.02)
Overweight/obese			1.00*** (1.00, 1.00)	1.00 (0.97, 1.02)
Age of the mother at first birth (years)				
<=20 years				
20–29 years			1.01*** (1.01, 1.01)	1.02 (0.99, 1.04)
30–49 years			1.01*** (1.01, 1.01)	1.02 (0.97, 1.07)
Pregnancy wanted				
Wanted				
Unwanted			0.79*** (0.77, 0.82)	0.96* (0.93, 1.00)
Household and community characteristics				
Exposure to mass media				
No				
Any				1.040** (1.01, 1.07)
Distance to health facility				
No problem				
Problem				0.99 (0.97, 1.01)
Exposure to family planning messages				
Not exposed				
Exposed				1.05*** (1.03, 1.08)
Household members				
1–4				
5–8				1.01 (1.00, 1.03)
More than 8				0.96** (0.93, 0.99)
Household head				
Male				
Female				0.99 (0.97, 1.02)
Wealth index				
Poorest				
Poorer				0.99 (0.96, 1.02)
Middle				1.036* (1.01, 1.07)
Richer				1.035* (1.00, 1.07)
Richest				1.029 (0.99, 1.07)
Social caste				
SC/ST				
OBC				1.00 (0.98, 1.02)
Other				0.99 (0.95, 1.00)
Religion				
Hindu				
Muslim				0.98 (0.95, 1.01)
Christian				0.93*** (0.89, 0.96)
Other				0.927** (0.88, 0.97)
Residence				
Urban				
Rural				1.038*** (1.02, 1.06)
Model diagnostics				
AIC	23,462	24,095	24,128	16,425
BIC	23,780	24,350	24,240	17,403
Pseudo *R*^2^	0.11	0.07	0.04	0.17

*p < 0.05, **p < 0.01, ***p < 0.001.

Table 5 presents the Fairlie decomposition analysis showing the relative contribution of child, maternal, and household characteristics to differences in girl child vaccination coverage. When only child characteristics were included, birth order accounted for more than 100% of the observed gap, whereas other variables made minimal contributions. Although the decomposition results indicate that observable characteristics jointly accounted for 84% of the difference in vaccination between girl children in son-preferring and non-son-preferring households, this percentage should not be interpreted as a causal effect. Rather, it reflects the proportion of the observed statistical gap that can be attributed to the measured covariates under the specified model. Birth order accounted for the largest portion of the explained component (≈21%), followed by ANC visits (≈29%) and household wealth (≈21%). These estimates describe how much of the vaccination gap is associated with each factor, assuming other included variables remain constant; they do not imply that these variables caused the gap. The remaining 16% represents unexplained heterogeneity, likely due to unmeasured behavioral, cultural, or community-level influences, rather than an effect attributable to a single mechanism. Birth order and the number of ANC visits contributed approximately 21 and 29%, respectively. Furthermore, an additional contribution of 21% was observed from household wealth among the household and community-level factors.

**Table 5:** Fairlie decomposition analysis showing the contribution of child, maternal, and household characteristics to girl child vaccination.

Background characteristics	Child characteristics contribution			Child and women characteristics contribution			Child, women, and household characteristics contribution		
	Coefficient	SE	Percent contribution	Coefficient	SE	Percent contribution	Coefficient	SE	Percent contribution
Child characteristics									
Age of the child	0.0001	0.00018	0.73	0.0006	0.00027	1.85	0.0007	0.00030	1.90
Birth order	0.0136	0.00181	103.35	0.0106	0.00303	30.91	0.0078	0.00306	20.76
Size of the child at birth	−0.0004	0.00033	−3.22	0.0000	0.00034	−0.02	0.0000	0.00037	−0.13
Birth weight	−0.0001	0.00008	−0.53	0.0004	0.00018	1.04	0.0006	0.00027	1.53
Maternal characteristics									
Women’s age				−0.0014	0.00130	−3.99	−0.0012	0.00111	−3.11
Women’s education				0.0099	0.00242	28.68	0.0041	0.00275	10.93
ANC visits				0.0100	0.00110	28.99	0.0108	0.00135	28.67
Place of delivery				0.0025	0.00067	7.31	0.0016	0.00074	4.16
Maternal BMI				0.0001	0.00056	0.35	−0.0001	0.00065	−0.15
Age of the mother at first birth				0.0012	0.00080	3.48	0.0011	0.00100	2.83
Pregnancy wanted				0.0006	0.00025	1.64	0.0009	0.00033	2.50
Household and community characteristics									
Exposure to mass media							0.0034	0.00162	9.08
Distance to health facility							0.0011	0.00063	2.96
Exposure to family planning messages							0.0035	0.00080	9.40
Household members							0.0003	0.00081	0.76
Household head							0.0000	0.00010	0.01
Wealth index							0.0079	0.00283	20.99
Social caste							−0.0009	0.00101	−2.39
Religion							−0.0007	0.00060	−1.75
Residence							−0.0035	0.00122	−9.32
Explain	**27.96**			**81.94**			**84.24**		
Unexplained	**72.04**			**18.06**			**15.76**		

## Discussion

This study aimed to investigate the relationship between maternal son preference and disparities in childhood vaccination coverage for girls in India, using nationally representative NFHS-5 data for girls aged 12–23 months. Our primary objective was to determine whether girls in households with son preference are less likely to be fully vaccinated and to identify the mediating factors driving this disparity. We applied a generalized linear model (GLM) to estimate relative risks and used Fairlie decomposition analysis to quantify how much child, maternal, and household characteristics explain the observed vaccination gap. The main finding is that girls in son-preferring households have significantly lower vaccination rates. The adjusted model did not show a statistically significant association, although the direction remained negative, suggesting that son preference acts as an underlying structural bias. This disadvantage is largely mediated by factors such as higher birth order, lower maternal education, fewer ANC visits, and household poverty, which together explain 84% of the gap, leaving a critical 16% of the variance unexplained by measured covariates.

Girls of fourth or higher birth order faced a 6% lower vaccination rate because they are typically raised in poorer, larger, and resource-constrained households, where parental time, health spending, and mobility for clinic visits are increasingly limited. In households with strong son preference, parents often prioritize immunization for earlier-born children, and this tendency may intensify when the desired gender composition has not yet been achieved. As a result, later-born girls are perceived as a continuation of fertility and receive reduced emotional investment and preventive care, increasing their risk of incomplete immunization (34). Mothers who attend more than three ANC visits achieve 9% higher vaccination coverage for their daughters, as they receive repeated information on vaccine schedules, timely vaccination, and the risks of preventable diseases. During these visits, ANMs, ASHAs, nurses, and doctors build trust by providing immunization cards, updating due dates, and giving clear guidance. These regular interactions and reminders improve maternal health-seeking behavior and support complete vaccination for girls, even in settings where misinformation or gender bias can limit care (35–37).

Institutional delivery increases girls’ vaccination coverage by 5% because newborn girls receive their first vaccines, such as BCG, before discharge, ensuring that the immunization schedule starts on time. Nurses, ANMs, and doctors also explain the next vaccine dates and required visits, enhancing mothers’ awareness. Institutional deliveries ensure that the Mother and Child Protection (MCP) card is properly completed, helping families remember due dates, while ASHAs and Anganwadi workers can track the child through the recorded information and conduct home visits to support vaccination completion. Educated mothers have a higher likelihood of ensuring complete vaccination for their daughters because they are better able to understand vaccine schedules, disease risks, and health messages, all of which support timely vaccination (38). They also have greater autonomy and decision-making power, enabling them to use the MCP card effectively and return for scheduled doses (39). In addition, education weakens harmful gender norms that undervalue girls, promoting more equal care and improving vaccination coverage (35, 40, 41).

Previous studies have concluded that unwanted pregnancy is not associated with reduced childhood vaccination (42). However, unwanted births, if the child is a daughter, are at a higher risk of incomplete immunization because they often occur among high-parity mothers with fewer ANC visits. Limited support and social judgment further reduce maternal engagement with health services (43). Such pregnancies are linked to lower emotional investment and weaker motivation for preventive childcare. In households with strong son preference, an unwanted girl child is more vulnerable to selective neglect, resulting in lower vaccination coverage (44). Exposure to media strengthens mothers’ autonomy in decision-making by offering clear, repeated information about vaccine benefits, schedules, and the risks of preventable diseases. It also helps counter myths and misinformation, which is particularly important in settings where rumours, low literacy, or gender bias may discourage vaccination of girls. Consequently, mothers become more confident in prioritizing preventive care and ensuring complete immunization for their daughters (45).

Rural residence, unexpectedly, showed a positive association. Previous studies did not find any significant difference (36, 46). In India, son-preferring households with girl children may also reflect the impact of targeted government programs such as Mission Indradhanush, which addressed barriers through door-to-door household listing, mobile vaccination teams, and, importantly, the engagement of non-health sectors—such as Anganwadi workers and community leaders—to build trust and dispel misinformation (47, 48). Conversely, larger household size remained a negative predictor. Larger household size and a higher number of under-five children reduce the likelihood of complete immunization, as confirmed by studies showing resource dilution, parental time constraints, and declining maternal attention with successive births (35, 49). Previous studies did not find any religious differences in childhood vaccination (50).

However, we found that children in Christian and other religious communities face a higher risk of incomplete vaccination, as these populations are concentrated in geographically isolated, tribal, hilly, or forested regions, such as the Northeast, where weaker health infrastructure limits access to ANC and routine immunization services. These hard-to-reach areas also have lower exposure to mass media, mobile networks, and government health campaigns. Moreover, dependence on forest-based livelihoods and irregular income patterns disrupt regular health visits, further increasing the risk of incomplete immunization (14). Although Christian children often benefit from higher maternal education, this does not translate into significantly higher immunization (33). Our findings further indicate a disadvantage for girl children in Christian households, where gender bias may override the benefits of maternal education.

The Fairlie decomposition analysis was used to quantify the relative contribution of these pathways. The analysis revealed that observable child, maternal, and household factors collectively explained 84% of the vaccination gap between girls in son-preferring and non-son-preferring households, leaving a significant 16% unexplained. This residual likely reflects the direct, internalized effects of son preference or other unmeasured socio-cultural factors such as community-level gender norms or the gender composition of siblings (11, 12). Among the explained portion, maternal factors were the most influential mediators. The number of ANC visits accounted for 29% of the gap, making it the single largest contributor. This highlights ANC as a critical intervention point, where healthcare systems can actively counteract gender bias by educating mothers on the equal importance of vaccinating all children (43). Household wealth index was the second-largest contributor (21%), highlighting the intersection of economic and gender-based disadvantage: poverty constrains access for all, but within poor households, girl children in son-preferring families are disproportionately affected (51). Maternal education explained 11% of the gap, reaffirming its role as a long-term, foundational investment in gender equity. A systematic review and meta-analysis found that maternal secondary or higher education increased the odds of full vaccination by 2.3 times (52). Notably, child-level factors, particularly birth order, explained 21% of the gap in the final model. While birth order initially explained over 100% of the gap in the child-only model (due to omitted variable bias), its independent contribution remained substantial, confirming that later-born girl children, especially in high-parity families, are at the highest risk of neglect (34). This decomposition provides a clear, evidence-based roadmap: to close the gender gap in vaccination, policies must prioritize increasing ANC utilization, improving female education, alleviating poverty, and specifically targeting high-birth-order families. The persistent 16% unexplained gap, however, serves as a stark reminder that technical interventions alone are insufficient; dismantling the deep-rooted cultural norm of son preference itself remains an essential, albeit more complex, long-term goal.

## Conclusion

This study demonstrates that son preference in India operates as a structural determinant of gender inequity in childhood vaccination, with girl children in son-preferring households exhibiting significantly lower full immunization rates. Although the direct association between son preference and vaccination became statistically non-significant after adjusting for socioeconomic and maternal factors, Fairlie decomposition analysis revealed that 84% of the observed gap was mediated through measurable pathways, most notably insufficient antenatal care (29%), higher birth order (21%), and household poverty (21%). These findings indicate that son preference does not act in isolation but is embedded within intersecting layers of disadvantage that cumulatively deprioritize later-born girls, especially in resource-constrained settings with low maternal education, limited media exposure, and unintended pregnancies.

Critically, a residual 16% of the disparity remained unexplained by observed covariates, likely reflecting unmeasured cultural norms, internalized gender bias, or community-level patriarchal attitudes that persist irrespective of socioeconomic status. This unexplained component underscores a fundamental limitation of purely supply-side health interventions: equitable vaccine coverage cannot be achieved without directly addressing the sociocultural mechanisms that render girls less visible in caregiving decisions. Therefore, effective policy must go beyond expanding service access to include gender-transformative strategies within existing health platforms. Antenatal care visits—already identified as the single largest mediating factor—should be leveraged to deliver explicit, evidence-based messaging regarding gender-equitable childcare, while Anganwadi workers and ASHAs should be equipped with training and protocols to identify and support high-risk households (e.g., fourth+ birth order, low ANC attendance). Moreover, routine immunization programs such as Intensified Mission Indradhanush should incorporate sex- and birth order-disaggregated monitoring and set equity-based performance targets. Closing the persistent 16% gap requires not only stronger health systems but also sustained engagement with the sociocultural roots of gender bias, positioning community health workers (CHWs) not merely as service providers but as agents of social change.

### Policy implications

The findings highlight that while the direct effect of son preference on girl children’s vaccination diminishes after adjusting for socioeconomic factors, it operates through mediating pathways, especially low ANC utilization, higher birth order, poverty, maternal education, and media exposure. This reveals a critical policy opportunity: antenatal care (ANC) visits and community health workers (CHWs) serve not merely as delivery points for clinical services but as powerful platforms for gender-transformative interventions. Given that >3 ANC visits explained 29% of the vaccination gap, every ANC contact must be leveraged to counteract son preference. Health providers can integrate gender-sensitive counselling emphasizing equal vaccine rights for all children. ANC platforms can also screen for high-risk profiles (e.g., fourth+ birth order, low maternal education, and socioeconomically disadvantaged mothers) and flag them for follow-up by ASHAs or Anganwadi workers.

CHWs, deeply embedded in communities, are uniquely positioned to challenge norms during home visits. Equipped with gender-aware training, they can reframe vaccination as a parental duty rather than a gendered choice and engage fathers and mothers-in-law, who often influence health decisions. In areas with entrenched son preference, such as eastern, central, and northern India, CHWs could use storytelling or community dialogues to shift attitudes, linking daughters’ health to family and national well-being. Policies should therefore mandate gender equity modules in ANC protocols and CHW curricula, supported by monitoring systems that track vaccination by sex and birth order. Programs such as Intensified Mission Indradhanush should explicitly include gender equity as a performance indicator. Closing the unexplained 16% gap requires more than improving access; it demands confronting bias where it persists—in households—through trusted local voices.

### Strengths and limitations

A key strength of this study is its foundation in the NFHS-5, India’s most comprehensive, nationally representative health survey. The large sample size of nearly 21,000 girl children aged 12–23 months provided exceptional statistical power, allowing us to detect even subtle disparities linked to son preference. Our use of advanced methods such as Fairlie decomposition is particularly valuable. It did not merely confirm that a gap exists but quantified exactly how much of it was driven by measurable factors, such as ANC visits (29%) or household wealth (21%), versus the persistent 16% that remained unexplained, likely rooted in deep-seated cultural bias. The sequential modeling approach further illuminated the pathway, showing how the direct effect of son preference was mediated through maternal and household decisions.

However, significant limitations remain. The Fairlie decomposition partitions statistical variation rather than causal pathways, and the 84% “explained” component reflects associations rather than deterministic effects. The remaining unexplained share may also include measurable factors not captured in the model, meaning interpretation should remain inferential rather than causal. The core measure of “son preference” is desire no sex composition of the child, which risks underestimation due to social desirability bias families may not admit to favoring sons, even anonymously. This could mean that our findings underestimate the true scale of the problem. The cross-sectional design is another constraint, as it captures a moment in time and cannot establish causality. Therefore, we cannot conclude that son preference causes lower vaccination—only that the two are associated. Furthermore, the data lack granular, real-world context. They do not capture the lived experience of an Anganwadi worker encountering a hesitant family or the specific reasons a fourth daughter’s vaccines were missed. These unmeasured community dynamics and interpersonal negotiations are likely embedded in that critical 16% unexplained gap, reminding us that behind every statistic lies a human story that our models cannot fully capture.

## Data Availability

Publicly available datasets were analyzed in this study. This data can be found at: https://www.dhsprogram.com/data/available-datasets.cfm.
